# Thermal Imager Range: Predictions, Expectations, and Reality

**DOI:** 10.3390/s19153313

**Published:** 2019-07-28

**Authors:** Dragana Perić, Branko Livada, Miroslav Perić, Saša Vujić

**Affiliations:** Vlatacom Institute, 11070 Belgrade, Serbia

**Keywords:** surveillance systems, thermal imaging, range prediction, minimal resolvable temperature difference (MRTD)

## Abstract

Imaging system range defines the maximal distance at which a selected object can be seen and perceived following surveillance task perception criteria. Thermal imagers play a key role in long-range surveillance systems due to the ability to form images during the day or night and in adverse weather conditions. The thermal imager range depends on imager design parameters, scene and transmission path properties. Imager range prediction is supported by theoretical models that provide the ability to check range performance, compare range performances for different systems, extend range prediction in field conditions, and support laboratory measurements related to range. A condensed review of the theoretical model’s genesis and capabilities is presented. We applied model-based performance calculation for several thermal imagers used in our long-range surveillance systems and compared the results with laboratory performance measurement results with the intention of providing the range prediction in selected field conditions. The key objective of the paper is to provide users with reliable data regarding expectations during a field mission.

## 1. Introduction

Long-range electro-optical surveillance systems [1] nowadays have wide and important application. The average user is looking for answers to two basic questions:

- How far can one see using a surveillance imager?
- How much shorter will the range be in bad weather (foggy or rainy conditions)?

Because of that, the key aspect of surveillance electro-optical imaging devices is the range at which an observer can solve a target acquisition task using the device. Typical acquisition tasks are the detection, recognition, and identification of relevant target objects, used to develop target visual perception criteria.

Imager range represents the maximal distance between the object of interest and the imager such that the observer is able to extract relevant data about the object using the generated images. The thermal imager range value depends strongly on the predefined visual perception task, and also on the observer’s training and knowledge.

In order to predict whether imagers meet the specified range performance requirements, or to compare the performance of different devices for intended applications, one can use field-based trials, laboratory measurement methods, or theoretical models. Theoretical models are needed to make the connection between laboratory measurements and field trials.

Infrared radiation (IR) thermal imager technology is expensive but powerful, providing images during the day and night and in adverse weather conditions. The range of such imagers is a very important parameter that every user wants to know. This parameter is subjective, having a different level of importance for all participants involved in the IR thermal imager application chain.

Due to the high costs, and despite significant IR thermal imager capabilities, investors in technology want to be assured of reliable predicted data regarding the imaging sensor range before they invest in the manufacturing technology.

Users want to learn about the range before they buy and deploy the imagers for a field mission. Additionally, range data are important for mission planning purposes. IR thermal imager manufacturers need a clear idea of the range because superior range data would be one of the most important advantages over competitors. It is in a manufacturer’s interest to provide IR imager range data as reliably as possible. Improving the IR imager range could be a complicated design task and will therefore significantly increase the unit price. Because of the importance of the range, it is very useful to have good laboratory control procedures as a part of quality control.

Thermal imager range assessment is extensively treated through thermal imager theory [2,3,4,5,6,7,8,9,10] and model [11,12,13,14,15,16,17] development, followed by modeling and experimental evaluation of the environmental influences [18,19,20,21,22,23,24,25,26,27,28,29,30,31,32,33]. Experimental field trials [34] are poorly covered in the literature due to the confidentiality of data. The influence of atmospheric scattering and turbulence [35,36,37,38] are studied mainly regarding laser systems, but all data could be used for thermal imagers. Target signature studies and their corresponding environmental influences [39,40,41,42] contributed to a better understanding of the thermal imager range.

In this article we review the literature and current status of IR thermal imager range predictions. Also, we apply the available models to the selected thermal imagers used in our systems, followed by laboratory measurements of the same equipment, in order to compare results. The intention is to identify key electro-optical parameters and define how to use these data for thermal imager comparison and range prediction in field conditions.

In Section 2, a short review of the factors affecting thermal image generation and the existing theoretical (numerical) models is presented. In Section 3 the results of selected parameters of thermal imager laboratory measurements and related model application results are extended to thermal imager range prediction. In Section 4, through a discussion of the obtained results, we derive recommendations for how to use such results for thermal imager comparison and extension to an expected range in field conditions.

## 2. Thermal Imager Range Predictions

**Figure 1:**
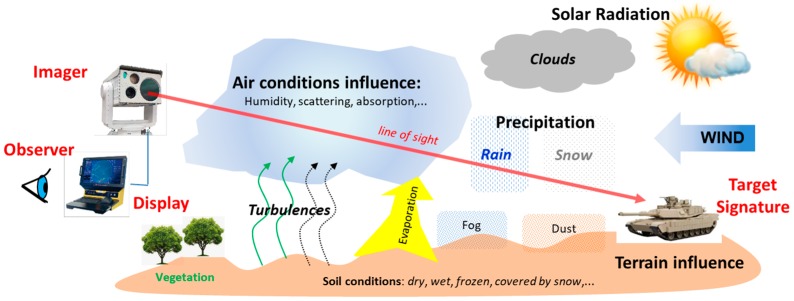
Image forming and perception influences.

Thermal imaging devices’ image formation process depends on many influencing factors, as illustrated in Figure 1 and listed in Table 1. The assessment of all listed influences is not possible with sufficient accuracy, especially in field conditions. That could lead to inaccuracy in the thermal imager range predictions.

As a first point, one needs to define the visual information discrimination criteria. In the case of surveillance systems where a human observer is involved, the level of visual data perception is a starting point. Because of that, it is very important that all involved have the same perception of the selected criteria to avoid any misunderstandings. In the case of automatic search and track systems, somewhat different criteria could be defined.

**Figure 2:**
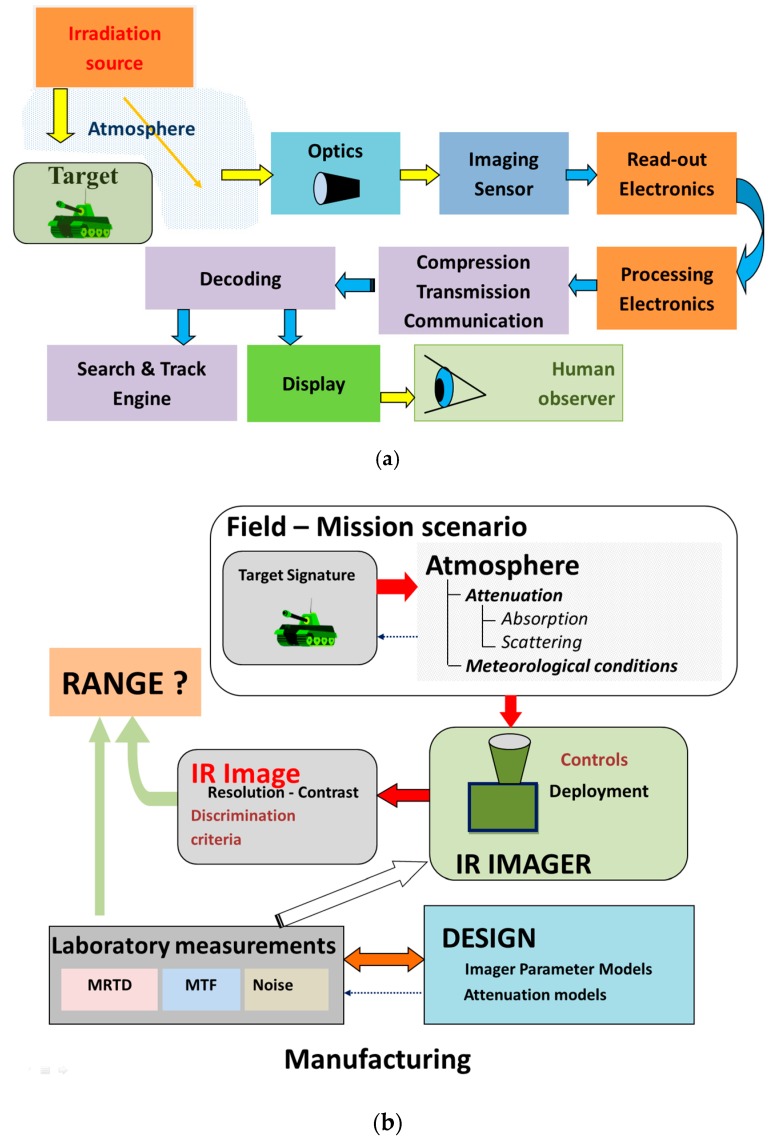
Imager range influences: (**a**) Image forming chain; (**b**) thermal imager range prediction data sources and their interconnection.

As a second point, infrared imager electro-optical properties related to IR scene image transformation are a limiting factor. The structure of the image forming chain is illustrated in Figure 2a and imager properties’ interconnection with other influences is illustrated in Figure 2b.

The thermal imager range is highly dependent on precipitation and atmospheric transmission conditions. The target signature (size, temperature difference of the target and background, etc.) is dependent on different factors.

It is impossible to give a simple answer to the question regarding the thermal imager range because the range can only be estimated with certainty if one has all the data necessary for analysis.

The thermal imager range for a selected target can be estimated only when all influencing factors are known. In field conditions, the determination of all factors is not always possible or they could be estimated with poor accuracy. Also, there is no universal theoretical model that can be applied following the data available in the field. Although various complex models have recently been developed for thermal imager analysis that can be useful in the technology planning and development phase, or in some specific mission, they cannot guarantee accurate results in field conditions.

In order to discern the target with a satisfactory image perception level, a thermal imager should have appropriate resolution and sensitivity to:

- Detect at specific (required) ranges
- Distinguish between targets and clutter (such as waves/surf zone, rocks, trees)
- Distinguish between targets and other false alarms (clutter, animals)
- Contrast and detail at longer ranges
- Distinguish details
- Distinguish behavior
- Distinguish friend from foe

Thermal imager electro-optical parameters could be theoretically analyzed (models) by knowing the basic parameters of the components used in the imaging chain. Also, they could be measured in the laboratory. Accordingly, model application is important. The process of visual data perception is hard to model because of observers’ individual differences. Because of that, observer models are still approximate and mainly involved in visual perception criteria or expressed by probability. So, we can determine only the probable thermal imager range according to the selected visual perception criteria.

**Figure 3:**
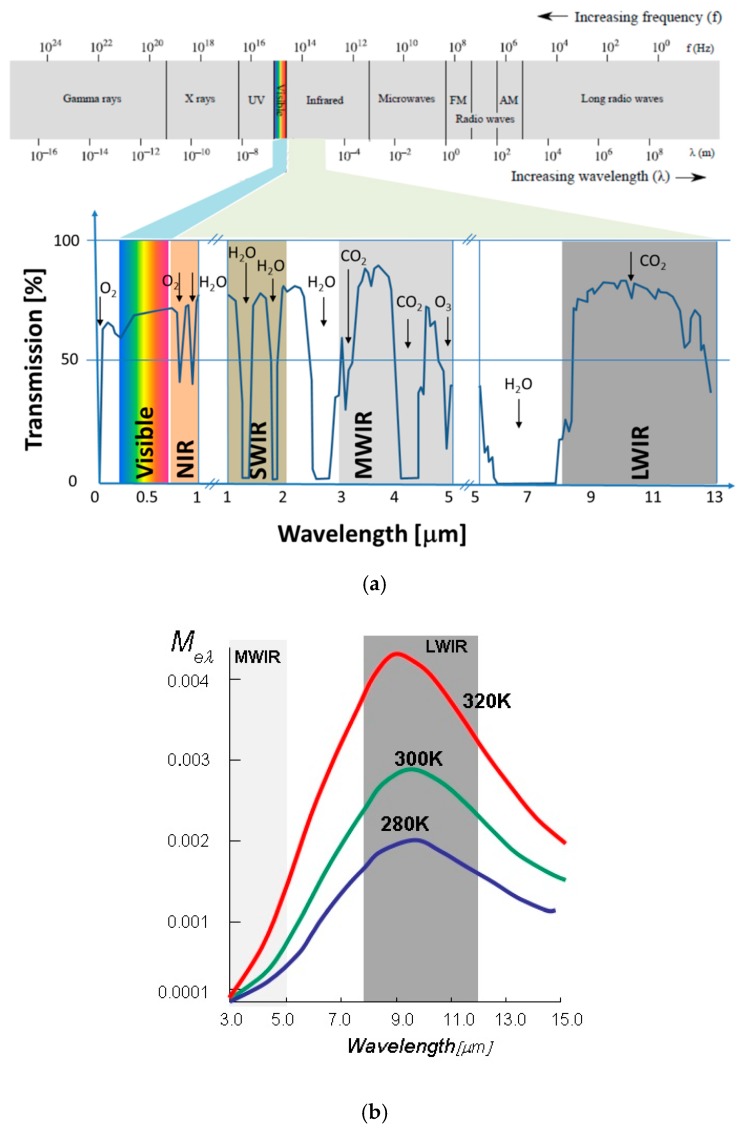
Thermal imager spectral band selection criteria: (**a**) Thermal image spectral sensitivity bands and atmospheric transmission windows; (**b**) blackbody exitance (around 300 K).

Thermal imagers are designed to use target radiation in the infrared part of the spectrum using so-called atmospheric transmission windows (either middle-wave infrared (MWIR) or long-wave infrared (LWIR)), as illustrated in Figure 3a. The spectral sensitivity band selection is important and has influence on the imager range because of the differences in scene IR signature and atmospheric transmission differences, as illustrated in Figure 3b and summarized in Table 2.

Scene (Target) Signature: The scene (target) IR signature represents the spatial distribution of the scene radiance that can be detected by an IR imager’s image sensor. The scene radiance has a complex structure due to the interaction between the target and background radiation properties (emissivity—ε, reflectivity—ρ, and temperature). The radiation scattering on the atmospheric transmission path also contributes to the scene’s overall signature and thermal contrast transformation. A thermal imager forms electronic images using only the apparent radiance difference. To simplify target signature description, it is common to use target apparent temperature difference, Δ*T*. This approach is supported by the fact that in laboratory conditions it is possible to achieve meteorological accuracy by controlling the target and background temperature. Target apparent (or equivalent) temperature difference, Δ*T*, can by defined as follows:

(1) $$\Delta T=T_{T}-T_{B},$$

where *T* and *T* represent the target and background apparent temperature, a proportional relationship given by Equations (2) and (3):

(2) $$T_{T}\propto \int_{\lambda_{1}}^{\lambda_{2}}\oint_{A_{T}}^{}\left[\varepsilon_{T}\left(x,y,\lambda \right)\cdot L_{T}\left(x,y,T\left(x,y\right)\right)+\rho_{T}\cdot M_{ES,t}\left(x,y,\lambda \right)\right]dxdyd\lambda ,$$

(3) $$T_{B}\propto \int_{\lambda_{1}}^{\lambda_{2}}\oint_{A_{B}}^{}\left[\varepsilon_{B}\left(x,y,\lambda \right)\cdot L_{B}\left(x,y,T\left(x,y\right)\right)+\rho_{B}\cdot M_{ES,b}\left(x,y,\lambda \right)\right]dxdyd\lambda ,$$

where *ε* represents the emissivity, *ρ* represents the reflectance, *L* represents the blackbody radiance, *T* represents the local temperature, and *M* represents the irradiation from external radiation sources. It is obvious that the target apparent temperature depends on a lot of factors that change over time, so there is no accurate way to determine them. For the purpose of modeling and measurement, it is widely accepted that Δ*T* = 2 K [43]. In the field application of the thermal imager, it is not possible to know what the target real temperature difference is.

Real thermal images are rich with details, but in the scene there are not a lot of thermally active elements, meaning that thermally the scene is in equilibrium, but the emissivity changes contribute significantly to target apparent temperature difference. It is calculated [44] that a 1% change of emissivity is equivalent to a 0.6 K temperature change in the MWIR spectral region or 0.9 K in the LWIR region.

Atmospheric Conditions: Atmospheric conditions, precipitation, and aerosol composition, which depend on the local climate and environmental conditions, have a complex influence on thermal imager range through transmission loss, target contrast loss along the transmission path due to scattering and turbulence, and influence on thermal processes in the scene and background. The atmospheric influences are presented in more detail in Section 2.5.

**Figure 4:**
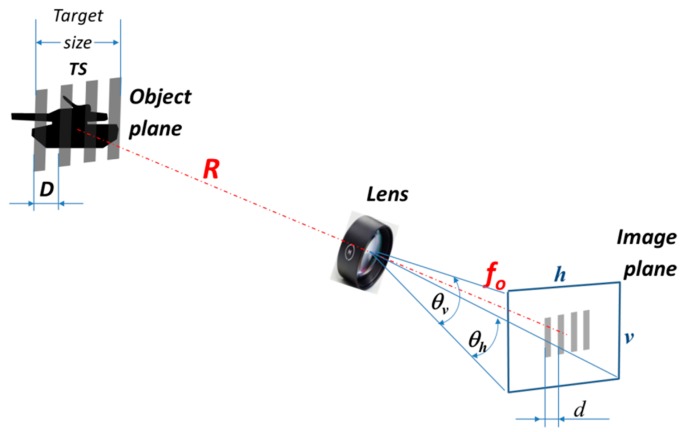
Spatial frequency concept.

Thermal Imager Properties: A thermal imager has a complex structure, as illustrated in Figure 2a, including an optical lens, image detector (FPA), image signal conditioning and processing electronics, image enhancement, and presentation (display) to prepare thermal images for perception by observers. The influence of all thermal imager components in the imaging chain could be theoretically analyzed using relevant models. To achieve commonality between theoretical models and laboratory measurements, several parameters (modulation transfer function—MTF, noise equivalent temperature difference—NETD [6], minimum resolvable temperature difference—MRTD) are selected to describe the thermal imager. In addition, these parameters could be used for thermal imager range prediction in field conditions. The models are mainly developed using linear system theory and scene and image properties description using spatial frequency concept illustrated in Figure 4.

Target (scene) radiance spatial distribution is described as modulation through line pairs (high, low) projected in the object plane.

The thermal imager model provides an analysis of how this modulation is transferred to the observer. Also, this concept provides a definition of the observer’s visual perception thresholds (Johnson’s criteria [45]) or a calculation of visual perception probability. There are several basic and important quantities that are used in the spatial frequency concept application. Equation (4) defines the imager’s instantaneous field of view (IFOV), Equation (5) defines the imager’s fundamental spatial frequency (Nyquist) *f*, and Equation (6) defines the relationship between the range and the target-related spatial frequencies R.

(4) $$IFOV=\tan^{-1}\left(\frac{\frac{d}{2}}{f_{0}}\right)=\tan^{-1}\left(\frac{p}{f_{0}}\right)=\tan^{-1}\left(\frac{\frac{D}{2}}{R}\right)\approx \frac{p}{f_{0}}\approx \frac{\frac{D}{2}}{R}\left[Radians\right],$$

(5) $$f_{N}^{*}=\frac{1}{2\cdot IFOV}=\frac{f_{o}}{2\cdot p},$$

(6) $$f=\frac{1}{\alpha_{T}}=\frac{1}{2\tan^{-1}\frac{\frac{TS}{N_{vp}}}{2\cdot R}}\approx \frac{N_{vp}\left[\frac{lp}{target}\right]\cdot R\left[km\right]}{TS\left[m\right]}\left[\frac{lp}{mRad}\right]\;R_{vp}\left[km\right]=\frac{TS\cdot f\left[\frac{lp}{mRad}\right]}{N_{vp}\left[\frac{lp}{target}\right]},$$

where *d* = 2*p* is the line size in the image plane defined by *p* pixel pitch; *f* is the lens focal length; *D* is the projected line pair size in the object plane; *R* is the distance between the target and the imager; *f* is the target-related spatial frequency, α is the target angular subtense, TD is the target size, and N is the number of line pairs per target size, related to selected visual perception criteria.

Starting from a basic radiometric relationship, the so-called “camera equation,” and applying the equivalent apparent temperature difference at the detector, one can obtain:

(7) $$\Delta T_{d}=\Delta T_{T}\cdot \tau_{a}\left(R\right)\cdot \tau_{o}\cdot \frac{A_{o}}{R^{2}}\cdot S_{o}\approx NETD\cdot SNR\to R^{2}=\frac{\Delta T_{T}\cdot \tau_{a}\left(R\right)\cdot \tau_{o}\cdot A_{o}\cdot S_{o}\cdot }{NETD\cdot SNR},$$

where *S* is the lens optical efficacy factor representing mainly aberration and diffraction losses, *τ* (*R*) represents atmospheric transmission losses along the line of sight, *τ* is the lens transmission, *A* is the lens aperture, and NETD and SNR represent the thermal imager noise and signal transformation parameters, respectively. Equation (7) cannot be used for finding an analytical range value solution, but it is a basis for the formulation of the computation models.

At the same time, to discern a target according to the visual perception level it is necessary to provide:

(8) $$\Delta T=\Delta T_{T}\cdot \tau_{a}\left(R\right)\geq MRTD\left(f\right)$$

(9) $$\frac{TS}{2\cdot N_{vp}\cdot R}\geq IFOV$$

Equation (8) expresses the requirement that the target apparent temperature difference should be higher than the thermal imager minimal resolvable temperature difference. This equation is used for thermal imager range prediction in some numerical models, and thermal imager range prediction following laboratory MRTD measurements.

Equation (9) expresses the requirement that target angular spreading should be in accordance with the thermal imager IFOV to be perceived, and define maximal thermal imager range for predefined visual perception task, due to geometrical resolution:

(10) $$R_{vp}^{G}=\frac{TS}{2\cdot N_{vp}\cdot IFOV}=\frac{TS\cdot f_{0}}{2\cdot N_{vp}\cdot p}.$$

This thermal imager range value represents the maximal range that could be achieved in ideal conditions, without atmospheric transmission loss and no video signal (contrast) loss in the thermal imager.

Display and Observer: Display, observer properties, viewing conditions, and visual integration time have a significant influence on the target image perception process. Display and human eye physical properties could be described in a model, but the viewing psychophysiological process is not easy to model. The advanced models introduce improvements in the perception process model to achieve more reliable prediction results. Due to observers’ individual differences, the probability of realization of the visual task is the best result that calculation could achieve.

### 2.1. Johnson’s Criteria

Johnson’s criteria were defined in the period 1957–1958, during his work on the definition image quality criteria for image inverter—intensifier tube-based devices [45]. He based these criteria on the work of previous researchers such as television pioneer Otto H. Schade, who developed mathematical tools for measuring the optical performance of television and fil cameras [46,47]. Johnson intended to measure the probability of object detection with the assumed resolution. Later researchers continued to improve his original criteria, but the basic concept is still valid [48,49].

Following the results from experimental research on visual data perception by human observers, Johnson determined the number of periodic line pairs over a target critical dimension that provide a related level of visual perception (detection, orientation, recognition, and identification). His results were accepted as an industry standard and are still used widely in the industry. During their usage they were slightly improved, with the most efforts being directed towards imaging channel theoretical models.

The spatial frequencies’ mean values for different visual data perception levels, as defined by Johnson’s criteria, are listed in Table 3. These criteria are derived for a visual data transformation process that keeps the contrast ratio in basic and transformed images; the perception level does not depend on the signal to noise ratio, assuming 50% perception probability.

These test patterns are used in field testing by application of specially designed mock-ups. The basic target dimension selected is 2.3 m, a standard tank size. During laboratory testing, test patterns are placed in the collimator focal plane and projected toward the thermal imager.

**Figure 5:**
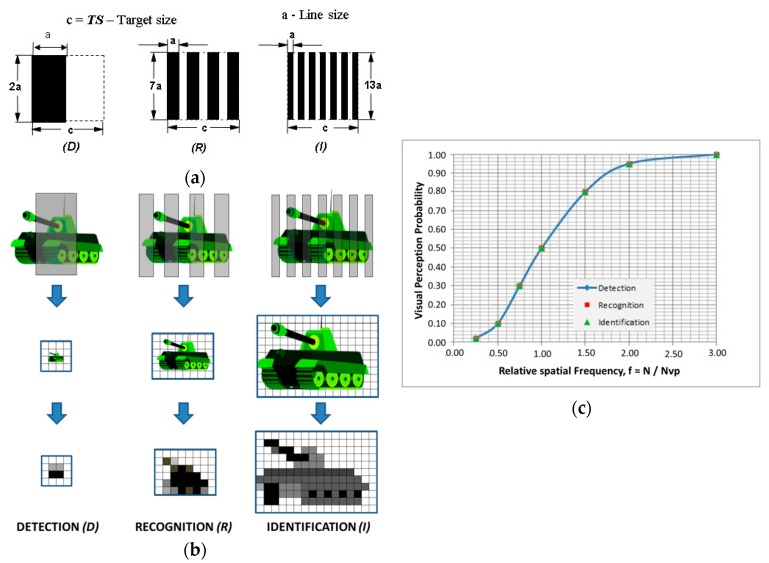
Commonly used test target patterns: (**a**) following D, R, I visual perception criteria; (**b**) illustration of application on a real target and anticipated image; (**c**) visual perception probability versus relative target spatial frequency.

In cases where the contrast changes during transformation, one must consider the visual data perception probability. The visual data perception probability for a defined signal to noise ratio is presented in Table 4, more details regarding this relation can be found in [12,13]. Also, the target size critical spatial frequency is related to the probability of visual perception, as illustrated in Figure 5c.

Following Johnson’s criteria, and taking note of practical needs for objective and combined laboratory and field testing, a set of standard test patterns is designed as illustrated in Figure 5a. Standard test patterns are designed for different levels of visual perception. The application of test patterns to a real target is illustrated in Figure 5b.

The success of the application of Johnson’s criteria, or any more advanced target visual perception and range prediction model, is strongly dependent on the level of significant factors of influence knowledge. Some typical inaccuracies introduced by selected factors are presented in Table 5, more examples can be found in [47].

### 2.2. Targeting Task Performance—TTP Metrics

All of the computer models based on the application of Johnson’s criteria deal with an isolated target having constant target contrast and a simplified human observer model. The advancement in thermal imager technology delivers more sensitive image sensors, providing better resolution. The first step in model advancement was to use a Barten human vision model [50,51] to introduce more accurate human vision modeling [52]. The second step was to calculate the probability of a specific target acquisition task that was a basis of the target task probability (TTP) approach [15,17,52,53]. The TTP metric extends target discrimination probability over all spatial frequencies:

(11) $$P=\frac{{\left(\frac{N_{resolved}}{V_{50}}\right)}^{E}}{1+{\left(\frac{N_{resolved}}{V_{50}}\right)}^{E}},$$

where $=1.51+0.24\cdot \frac{N_{resolved}}{V_{50}}$, *V* is a value of the metric necessary to perform a task 50% of time that should be determined experimentally for a predefined class of objects, and *N* is the number of pixels over the target at the display device. This approach provides more accurate data for target acquisition task probability. For the purpose of surveillance system range prediction, Johnson’s 50% probability-based criteria are still applicable.

### 2.3. Thermal Imager Modeling and Range Prediction

Thermal imager model accuracy makes the lowest contribution to the overall inaccuracy, so improving the model accuracy does not significantly improve the accuracy of thermal imager range prediction in field conditions. Thermal imager models are mainly aimed at supporting thermal imager analysis and synthesis through the design process. Advanced models improve visual task probability determination and the overall thermal image quality increases. Therefore, it is important to have more accurate models to support thermal imager design.

During the last 50 years thermal imager models have been constantly improving. The first and best known, the thermal imager static performance model [12], provided a model that was successfully used for thermal imager design. Later, it was improved, introducing changes suitable for sampled imagers and focal plane array-based imagers, and transformed to FLIR92 [54,55] and the NVTherm model [13], the most commonly commercially available and widely used thermal imager model, which incorporates TTP-related calculations, too. There are several other models that have been developed around the world [56,57,58,59,60,61,62,63,64,65]. A model based on perceived temperature difference [14] was successfully integrated within NVTherm [16].

One of the first improvements was the introduction of an improved model for human visual system—eye transfer function [52] using results from Barten’s eye sensitivity model [51]. This was followed by an improved detector and optics model [66]. Further improvements included better processing electronics and detector noise modeling suitable for FPA applications [67].

Several simplified models suitable for fast estimation of thermal imager parameters [68,69,70] are useful for understanding the basic physical processes used for thermal image formation.

A condensed review of the thermal imaging system model development path [67,71,72] shows that over a 40-year period the models were significantly improved and proved an important tool for system analysis and synthesis.

#### 2.3.1. Modulation Transfer Function (MTF)

An electro-optical system model is defined using linear systems theory. The spatial pulse response of the system is Fourier transformed into a spatial-frequency optical transfer function. Instead of spot size, we consider a frequency response that facilitates additional insight into the behavior of an imaging system, particularly in common situations where several subsystems are combined.

Using the spatial-frequency domain, we can multiply the individual transfer function of each subsystem to give the overall transfer function. This approach has the ability to identify the origin of the performance limitations and which crucial components must be redesigned in order to improve the overall image quality [73,74].

Since the shape of the MTF function is known for square detector, diffraction-limited optics etc., system MTF function can be roughly predicted in terms of limitations and maximum achievable resolvable spatial frequency, which defines the resolution of the system.

Computer software calculates MTF functions based on input parameters. One of the widely used computer software for electro-optical simulations is NVThermIP [11], which models different types of thermal imagers that operate in the MWIR and LWIR spectral bands. The NVThermIP predicts the contrast threshold function (CTF) of the system, and uses CTF to predict the target acquisition range performance likely to be achieved using the sensor. The model also predicts the minimum resolvable temperature difference (MRTD) of the system. Each subsystem is modeled using its MTF function, and system MTF is obtained as the product. Johnson criteria are implemented in the model, but other target parameters and criteria can be taken into account.

Thermal imaging system MTF function can be measured in the laboratory and is suitable for the analysis of system design success. Also, the MTF analysis could help to indicate the root cause of deficiencies in case a system fails to perform as expected.

#### 2.3.2. Minimal Resolvable Temperature Difference (MRTD)

The MRTD provides the connection between Johnson’s concept of resolvable bars across the target critical dimension and thermal imager system performance. The MRTD measurement that includes the observer could be routinely carried out in the laboratory with good accuracy and repeatability. Also, MRTD provides a reasonable connection between the thermal imager model and imager capability to provide the expected range in field conditions. Following a basic thermal imager model [12], *MRTD* (*f*) can be calculated as follows:

(12) $$MRTD\left(f\right)=\frac{SNR\cdot NETD}{MTF\left(f\right)}\cdot F_{TIS}\left(f,DP\right)$$

where *f* is the spatial frequency; *SNR* is the target signal to noise ratio; *MTF*(*f*) is the thermal imager total modulation transfer function; *NETD* is the noise equivalent temperature difference; and *F* (*f*, *DP*) is the thermal imager-related design function depending on system design parameters (DP). This MRTD model tends to be optimistic at low spatial frequencies and pessimistic at higher spatial frequencies, but is a thermal imager model that can be proven through laboratory measurements and used for thermal imager range performance estimation [75].

Thermal imager performance models developed using only Johnson’s criteria cannot be effectively applied to thermal imagers using FPA (focal plane array) detector and digital image processing technology, for two reasons:

(1) The Johnson metric is based on the system response at a single frequency, so it does not cover the effect of change of the image frequency spectrum through digital processing,
(2) Johnson criteria-based models do not cover image effects below the limiting frequency that is used in modern digital imaging systems to increase image quality.

Despite the listed deficiencies of Johnson metrics, it has been successfully applied for 30 years, and is still useful for simplified predictions. It can still be successfully applied for thermal imager range prediction in field conditions [76].

Thermal imager technology development provides high-quality thermal imagers that can deliver a higher level of target acquisition performance, but cannot override the basic limitations related to atmospheric transmission losses. In cases where the target range allows target images to be generated, further improvements provided by models could lead to better target acquisition [77].

#### 2.3.3. Noise-Equivalent Temperature Difference (NETD)

The noise-equivalent temperature difference (NETD) is the smallest measurable signal produced by a large target [6]. These data represent system sensitivity. Types of noise that affect an imaging system include photon noise, detector-related electronic noise such as Johnson noise, 1/f noise, processing electronic noise such as amplifier and MUX noise, and fixed-pattern noise. Some of these sources can be lowered in the modern FPA, except for random noise. Background-limited performance (BLIP) means that the random noise is present only because of photon detection process fluctuations. The analytical form for NETD can be derived as Δ*T* from the signal to noise ratio equation:

(13) $$NETD_{BLIP}=\frac{4\cdot F^{2}\cdot \langle n_{sys}\rangle }{A_{d}\cdot t_{int}\cdot \int_{\lambda_{1}}^{\lambda_{2}}R_{q}\left(\lambda \right)\cdot \frac{\partial M_{q}\left(\lambda ,\;T_{B}\right)}{\partial T}\cdot \tau_{optics}\left(\lambda \right)d\lambda }\cdot$$

BLIP noise limitation is very important for detector design and FPA field of view or F# definition.

For an advanced digital IR imager using focal plane array (FPA) detectors, a new 3D system noise concept is adopted for system image noise analysis. Complex 3D (time, vertical, horizontal) noise parameters are derived through statistical analysis of the consecutive digital image datasets while a system is viewing a constant background stimulus. The 3D noise calculation model calculates statistical variations (standard deviation or variance) of image data along selected dimensions, as defined by noise type through the whole pixel stream.

### 2.4. Thermal Imager Laboratory Performance Measurements and Range Prediction

Using well-known thermal imager laboratory measurement methods [78,79] and well-configured measurement equipment [80], one is able to access key parameters such as signal transfer function (SiTF), NETD, MRTD, MTF, and imager limiting resolution (USAF 1956 test chart). These parameters provide a basic set of data to assess the imager’s quality. Using the standardized MRTD measurement procedure and measurement data processing [43,81] one can predict the thermal imager range for a selected target and field atmospheric attenuation factor. A standard processing procedure is defined for the target and atmospheric conditions that seem most probable in field applications and provides accuracy depending on accuracy of measurements [82,83]. Also, a similar measurement procedure could be developed and applied for advanced imagers [84], and the measurement results could be processed according to the TTP concept.

### 2.5. A Short Review of Atmospheric Influences on Thermal Imager Range Predictions

Atmospheric optics is important in areas such as free air optical communication, high-energy laser propagation, space remote sensing and observation systems, and space laser communication. There have thus been a lot of studies in this area [22,24,25,30,85,86,87]; they go into more detail than is necessary for studies of the atmospheric influences on imaging, but could be applied to imaging process analysis. Visible imaging through the atmosphere is reviewed in [19,20,21], and IR imaging is analyzed in [88,89,90]. Adverse weather influences in marine conditions are analyzed in [90,91,92,93] for maritime conditions, desert conditions [94], and heavy fog [91]. Atmospheric transmission influence on IR temperature measurements is presented in [26,28], and thermal imager application in civil aviation [95].

Imaging systems are designed to deliver the best performance in good weather conditions [96]. In reality, bad weather cannot be avoided. The prediction of the influence of weather conditions on IR imager application in field conditions is not accurate enough because it is not possible to know all necessary data along the imaging trace and because of atmospheric transmission’s dependence on the target range. The key thing to have in mind is that the image could be worse than expected in the case of really bad weather. The good news is that weather influence parameters, used in common modeling cases or in laboratory predictions, are applicable to most situations during a field mission.

**Figure 6:**
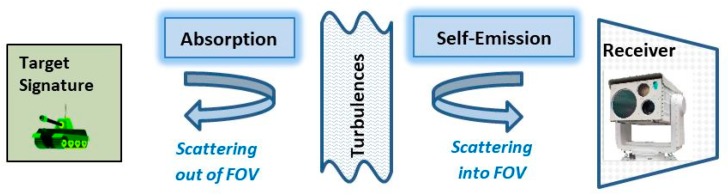
Atmospheric processes influencing attenuation coefficient and thermal imager range.

Atmosphere introduces changes to the imaging signal by several means that limit the range of the imager, affecting radiation propagation through the atmosphere. The major physical phenomena that affect electromagnetic radiation transfer are illustrated in Figure 6.

Atmospheric attenuation/extinction is the total reduction of radiation along the line of sight, and includes absorption and scattering [6]. The spectral transmittance is determined using the Beer-Lambert law. Extinction depends on all atmospheric constituents (aerosols, fog, rain, snow). It is expressed as the parameter *τ* (*λ*) in the Beer-Lambert equation:

(14) $$\tau_{ATM}\left(\lambda ,\;R\right)=e^{-\sigma \left(\lambda \right)\cdot R}$$

(15) $$\sigma \left(\lambda \right)=\sigma_{abs}\left(\lambda \right)+\sigma_{sc}\left(\lambda \right),$$

where *σ*(*λ*) is the total attenuation coefficient; *σ*(*λ*) is the attenuation due to absorption (gases, molecules) and *σ*(*λ*) is the attenuation due to scattering (particles).

Atmospheric transmission models are fairly complicated and use a huge database, so they are suitable for dedicated calculations and analysis [97,98]. The average user in the field cannot use these calculations, so need simplified methods based on locally available meteorological conditions-related parameters that have a key influence on atmospheric transmission: meteorological visibility and air humidity.

Meteorological visibility is the greatest distance in a given direction at which it is possible to see and identify with the unaided eye. Using 2% as the limiting human eye sensitivity, Koschmieder’s law defines the connection between the mean value of the atmospheric attenuation coefficient *σ* in the visible part of the spectrum (λ = 0.55 μm) and the meteorological range *R*:

(16) $$R_{v}=\frac{\ln0.02}{\sigma_{v}}=\left|\frac{3.912}{\sigma_{v}}\right|,\;\left[km\right]\to \sigma_{v}=R_{v}=\frac{\ln0.02}{\sigma_{v}}=\frac{3.912}{R_{v}},\;\left[km^{-1}\right].$$

In case the meteorological range is measured by a transmissometer, the scattering attenuation factor in the IR spectral range is calculated for the continental climate [92]:

(17) $$\sigma_{sc}=3.91\cdot {\left(\frac{\lambda_{m}}{0.55}\right)}^{-q},\;q=0.585{\left(R_{v}\right)}^{\frac{1}{3}},$$

where *λ* is the spectral band central wavelength (λ = 4 μm, MWIR band, and λ = 10 μm, LWIR band). The absorption attenuation coefficient depends on the water vapor content on the transmission path. This type of approximation is applicable for a predefined climate type, so a similar approximation could be derived for other climate types using a similar approach.

Absorption and scattering contributes to a reduction in the amount of radiation that reaches a sensor. Scattering and turbulence have an influence on the radiation changes generated along the line of sight, resulting in image blurring and a loss of detail.

Scattering on the transmission path has an influence on atmospheric MTF and contributes to IR image degradation. Studies of the scattering influence on MTF [94] could be used in the development process of digital filters for image de-hazing.

Turbulence (heat haze) cause image blur-related degradation [35,36,37,38] that could be represented as the turbulence component of the atmospheric MTF. This approach is not exact for atmospheric modeling, but provides a first-order approximation [91]. A limitation of this approach is that the MTF theory is based on linear time-invariant processes. Turbulence is not necessarily uniform across an image, but is often assumed to be so for modeling purposes.

A key application of the atmospheric MTF function is in the development of digital image processing filters as compensation for atmospheric-related blur. The application of polarization to atmospheric blur techniques has also been studied as an image improvement technique [99,100,101].

The weather’s influence on an imager’s range is very important in some applications (i.e., civil aviation [102]) and very specific in maritime and tropical weather conditions [90,95]. Therefore, apart from modeling, it is important to collect and study experimental data in order to provide better mission planning.

## 3. Results

In order to validate and compare various methods for range prediction (calculations and experimental measurements), we provided three multi-sensor imaging systems that have different types of thermal imaging sensors (two MWIR sensors with different resolution and one LWIR sensor). All three multi-sensor systems were developed by Vlatacom Institute, Belgrade, Serbia for border protection projects.

A set of basic technical specifications that are necessary as input for the calculation of range and target spatial frequencies selection is presented in Table 6.

As it is complicated to determine the exact composition of atmosphere over the path of interest, an engineering approach is needed to create a model that is applicable in various weather conditions and can be validated in EO lab and field tests. MODTRAN (MODerate resolution atmospheric TRANsmission) is one of the four codes that were created, and defines several representative geographical and seasonal models (Tropical, Mid-latitude winter, Mid-latitude summer, Sub-arctic winter, Sub-arctic summer, U.S. standard) and aerosol models (Rural, Maritime, Urban, Desert, Troposphere, Navy Aerosol, Radiative fog, Advection fog). The MODTRAN computer code is used worldwide by research scientists in government agencies, commercial organizations, and educational institutions for the prediction and analysis of optical measurements through the atmosphere. The key to the model’s accuracy is the completeness of the database for spatial particle and molecule distributions on the trace.

In this paper we analyze how imager ranges vary with specific atmospheric attenuation, and explore the parameters contained in a database for a specific geographical area. This will be achieved using the software packages MODTRAN, Spectral Sciences, Inc., Burlington, MA, USA and NVThermIP, U.S. Army Night Vision and Electronic Sensors Directorate, Fort Belvoir, VA, USA.

**Figure 7:**
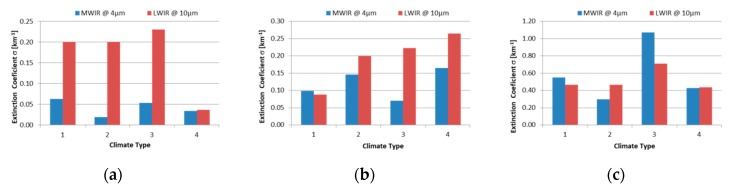
Atmospheric attenuation coefficient’s dependence on meteorological visibility, climate type (1—Mid-latitude summer urban; 2—Tropical desert, 3—Tropical navy aerosol; 4—U.S. standard 1976 rural) and imager spectral sensitivity in the case of: (**a**) good visibility—23 km, (**b**) medium visibility—5 km, (**c**) bad visibility—1 km.

In order to obtain the specific atmospheric attenuation for each system, we calculated the atmospheric transmittance at the center of the MWIR spectral region (at 4 µm) and at the center of the LWIR spectral region (at 10 µm). Atmospheric transmittance is calculated for path length 1 km, using MODTRAN geometry configuration with sensor altitude 1 km and sensor zenith 180° (vertical transmission path). From these results, the extinction parameters are calculated and presented in Figure 7.

**Figure 8:**
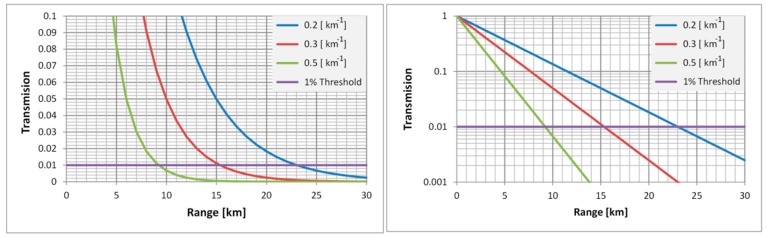
Atmospheric transmission value dependence on range and attenuation coefficient (**Left**: log-linear scale; **Right**: linear scale).

The atmospheric attenuation coefficient’s dependence on meteorological visibility and climate type, calculated using a MODTRAN code, is presented in Figure 7 for selected climate types and meteorological conditions, represented through meteorological visibility in the MWIR and LWIR part of spectrum. These values could be used to get an idea of the values of attenuation coefficients that could be expected in field conditions. The atmospheric transmission value’s dependence on range for three selected attenuation coefficient values, against the anticipated threshold transmission value of 1%, is presented in Figure 8, showing how the imager range changes when the attenuation coefficient (weather conditions) changes.

After the calculation of atmospheric attenuation coefficients for MWIR and LWIR spectral ranges, we proceeded with the calculation of ranges in given atmospheric conditions for all three multi-sensor imaging systems, SYS1, SYS2, and SYS3, which are presented in Table 6. The results of this calculation are presented in comparison to other calculation methods in Table 7.

**Figure 9:**
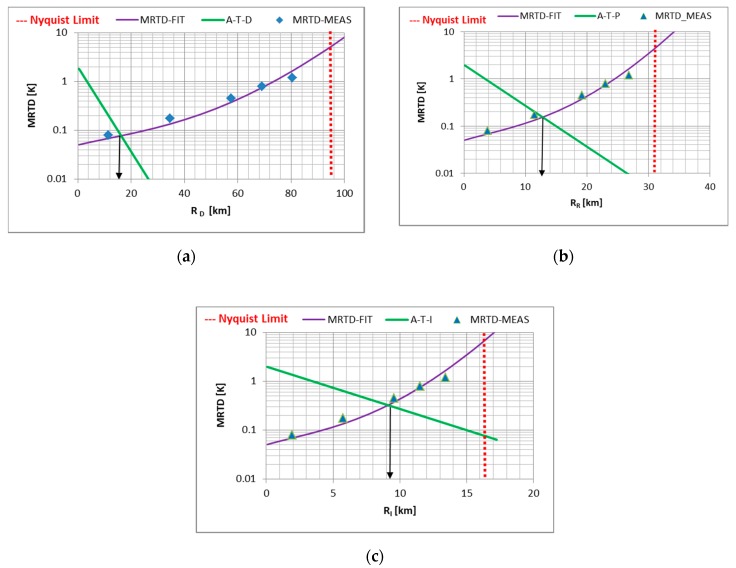
Imager range prediction from MRTD measurement results: (**a**) Detection—D; (**b**) recognition—R; (**c**) identification—I.

In our electro-optical laboratory we use a CI Systems, Israel, IR 3000 mm collimator suitable for long-range imager measurements. In the case of the thermal imagers, we apply standard NATO STANAG 4347 and 4349 [43,81] methodology and procedures for laboratory-range predictions. Examples of the MRTD measurements and related standard processing for thermal imager range determination are presented in Figure 9.

The thermal imager visual perception-related range data are summarized in Table 7.

Geometrical model-based range prediction is based on the following criteria: D—2 pixels per target size, R—7 pixels per target size, and I—13 pixels per target size.

Calculations with the NVThermIP model are performed for good viewing conditions (standard U.S. atmosphere with 23 km meteorological visibility when the atmospheric attenuation factor is 0.2 km^−1^), and for bad weather conditions (mid-latitude summer and tropical climate models with 5 km meteorological visibility when the atmospheric attenuation factor is 0.35 km^−1^).

Range prediction using laboratory MRTD measurements is determined using a target resolving task according to STANAG 4347: 50% probability for two target sizes (human and vehicle) and 2 K temperature difference between the target and the background (the atmospheric transmission value is determined using an atmospheric absorption coefficient value of 0.2 km^−1^, which is usually considered a good atmospheric condition.

**Figure 10:**
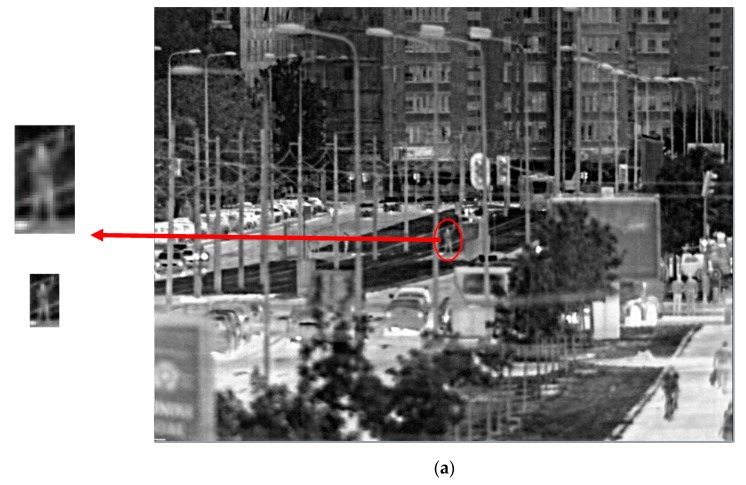

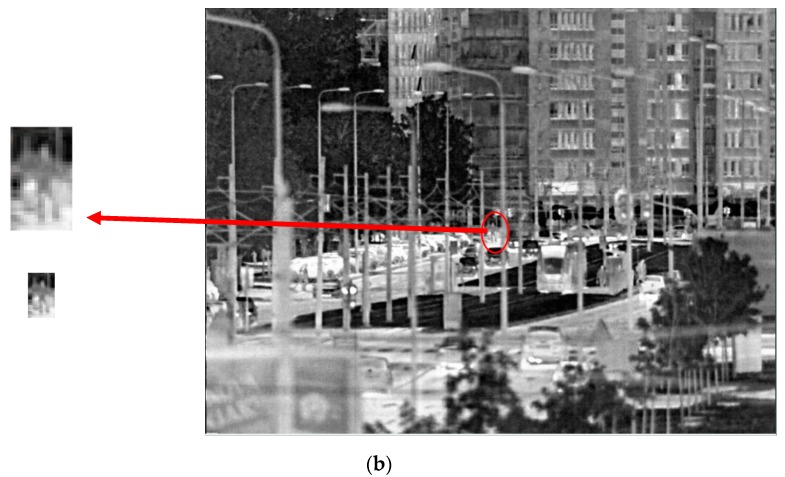
Images taken by SYS3 that represent human identification at a calculated range: (**a**) 800 m; (**b**) 1100 m.

For the purpose of illustrating the calculation results, Figure 10 presents images taken by SYS3 that show human identification obtained at 800 m and 1000 m, respectively. Images are taken in clear-sky, daytime conditions, with an external temperature of around 18 °C. On the right is the selected snapshot, and on the left is an enlarged image of the person of interest.

The structure of the scene presented in Figure 10 makes it hard to detect the object of interest, but once detected one can find that it is a standing male person (visual perception level—identification) with one hand up (a) and both hands open (b). The related ranges are selected in accordance with the predicted identification range values determined for SYS3 and human objects, as shown in Table 7.

## 4. Discussion

In accordance with the results given in Table 7, we could identify high-end surveillance systems as:

- Ultra-long range—ULR (represented by SYS 2)
- Long range—LR (represented by SYS 1)
- Medium range—MR (represented by SYS 3)

Detection is the first level of a surveillance system application task, but for fulfilling mission-related visual information perception tasks, identification is required. This means that the imager detection range is important but not sufficient for a mission’s success. The higher the detection range values, the higher probability there is of mission success.

The ULR and LR surveillance systems are diffraction-limited systems. Because of that, geometrical RDI range values that do not take this effect into account are more optimistic than model-based predicted values and laboratory measurement-based values. Therefore, it is not appropriate to use these values for mission planning purposes. Laboratory measurement-based values (see Table 7) are set between the model predicted values for standard and bad atmospheric conditions. Laboratory measurement-based range values could be the closest to the expected range during a surveillance mission and thus suitable for mission planning, but one must bear in mind that the field expected imager range could have high inaccuracy.

Analysis of the atmospheric attenuation coefficient dependence on meteorological visibility has shown that in poor-visibility maritime scenarios, values of the atmospheric transmittance in the MWIR spectral range are higher than in LWIR.

## 5. Conclusions

Modern imaging systems (both visible and infrared) are designed to have the best performance in clear weather conditions. All theoretical imager models consider such conditions, so that the predicted imager range is related to clear weather. However, these assumptions do not apply to real missions, and weather conditions change over time. Therefore, imagers’ range in field conditions is limited by weather influence.

Answering basic questions about the range of an imaging system is not simple and could even be impossible in cases where all mission-related data are unknown. Even when all data are known, the answers generated could have a high level of inaccuracy. We reviewed and referred to previous results confirming the importance of range prediction on the one hand and clarity of the data on the other hand. Most range prediction models deal with imager parameter definitions and their influence on the imager’s range, with reference to image perception tasks. These models provide us with tools for imager analysis and synthesis used to define the optimal design. To confirm the usefulness, their results are extended to imagers’ range prediction using different approaches such as visual information perception modeling, target IR signature, and atmospheric path-related image degradation.

Despite all the deficiencies, Johnson’s criteria are still applicable for IR thermal imager range prediction. IR thermal imager model developments provide very useful tools for analysis and synthesis, applicable during development for design optimization, but do not significantly improve imager range predictions in field conditions. An imager’s range prediction using a simple geometrical model based on Johnson’s criteria provides optimistic range data. IR thermal imager MRTD measurements, which are also based on Johnson’s criteria, deliver measurement results that at the same time provide a quality control method to assess how well the imager is manufactured, and data that could be processed to predict the imager’s range for predefined target and atmospheric conditions. Potential users should apply the predicted range data (geometrical and MRTD-based) to get an idea of the imager’s capabilities in field conditions. An IR imager cannot “see” farther than its geometrical range, and we should expect that most of the time it will only be able to “see” as far as the MRTD-based range. In the case of adverse weather conditions, the imager’s actual range will be less than the values. For an imager’s application in “bad” atmospheric conditions, a user should form and use their own database of experimental data for range reduction.

To predict imager range during a mission, one needs to carefully define the mission goals and related visual data perception criteria. The geometrical range is always highly optimistic, but those data are useful to judge the imager design and components’ capability. The second step is to study imagers’ laboratory measurement-based range values and compare them with the mission requirements. The third step is to apply a related model using the mission weather (environmental) profile. Neither of these steps will guarantee that the imager’s field range will be as predicted. The best way to improve the accuracy of the imager’s range estimation is to have one’s own database containing trackable weather conditions and the imager’s range during long-term application in the field.

## Figures and Tables

**Table 1:** Thermal image forming and perception influence factors.

Observer	Display	Thermal Imager	Platform	Atmosphere	Target
Training Motivation Experience Observation time Pre-briefing Stress Task Load Fatigue Age IQ Visual acuity	Screen size Color SNR Resolution Gray scale Persistence Response speed Ambient illumination Distance	Type Spectral band Field of view Resolution Dynamic range Sensitivity Operation mode Noise F# Diffraction	Field of regards Vibration Environment Speed Target exposure time Crew size and interaction Controllability	Visibility Clouds Sun Angle Range Absorption Scattering Temperature Precipitation Humidity Turbulence	Type Size Shape Clutter Camouflage Reflectance Emissivity Background temperature Contrast Motion

**Table 2:** Properties related to thermal imager spectral sensitivity band selection.

Spectral Band	SUN Irradiance [W/m^2^]	BB 300 K Exitance [W/m^2^]	Thermal Contrast ΔT/T	Absorption	Scattering
VIS	750	0	-	high	high
MWIR	24	1.5	0.39	lower	higher
LWIR	1.5	130	0.17	higher	lower

**Table 3:** Visual perception levels versus critical spatial frequency, as defined by Johnson’s criteria.

Critical Spatial Frequency	Visual Perception Level			
	Detection	Orientation	Recognition	Identification
Line pairs per target size	1 ± 0.25	1.4 ± 0.35	4 ± 0.8	6.4 ± 1.5
Pixels per target size	2	-	7	13

**Table 4:** Visual data perception probability and required minimal signal to noise value.

Visual Perception Probability	Signal/Noise Ratio
1.0	5.5
0.90	4.1
0.80	3.7
0.70	3.3
0.60	3.1
0.50	2.8
0.40	2.5
0.30	2.3

**Table 5:** Factors contributing to application of visual perception-based range estimation in the field.

Factor	Inaccuracy (%)	Comment
Scene signature	50 to 250	Clutter effects and density
Target properties	Up to 200	Daily and seasonal differences
Atmospheric conditions	100	Transmission, scattering and turbulences
Thermal imager parameters	30	Model accuracy and measurement errors
Display and observer	100	Ambient illumination and observer training
Observation time	200	Visual data integration and discrimination

**Table 6:** Technical specifications of the multi-sensor imaging systems used in the tests.

	SYS1	SYS2	SYS3
	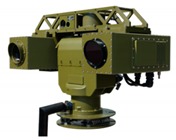	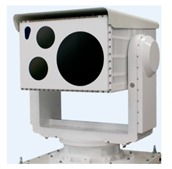	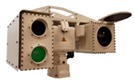
Spectral range	MWIR (3.6–4.2 μm)	MWIR (3.6–4.2 μm)	LWIR (8–12 μm)
Image sensor resolution	640 × 512	1280 × 1024	640 × 480
Pixel size	15 μm	15 μm	17 μm
Lens focal length	330 mm	1200 mm	225 mm
Lens aperture (F#)	4	4.7	1.5

**Table 7:** Thermal imager range prediction measurements for selected surveillance systems.

		Visual Perception Range (km)											
System Type	Target Type	Detection				Recognition				Identification			
		G	MS	MB	L	G	MS	MB	L	G	MS	MB	L
**SYS 1**	Human	13.9	9.1	7.12	8	3.5	2.5	2.4	2.6	1.8	1.7	1.4	1.2
	Vehicle	33.7	13.9	9.9	13.1	8.4	5.3	4.6	6.8	4.3	4.2	3.9	3.6
**SYS 2**	Human	50	18.1	11.3	13	12.7	8.8	6.3	7.6	6.5	5.5	5.3	4.6
	Vehicle	>50	22.4	13.5	16	30.7	13.6	9.1	12	15.3	12.2	8.4	9.2
**SYS 3**	Human	6.3	4.3	3.7	5.2	2.1	1.0	1.0	2.0	1.1	1.0	0.8	1
	Vehicle	15.2	7.6	5.9	10.5	5.1	2.3	2.1	4.5	2.6	1.8	1.8	2.3

G—geometric, MS—NVThermIP model, standard atmosphere (α—0.2); MB—NVThermIP model, bad atmospheric conditions (α—0.35); L—based on MRTD laboratory measurements and STANAG 4347 procedure. Human (size 1.8 m × 0.5 m, Δ*T* = 2 K); Vehicle (size 2.3 m × 2.3 m, Δ*T* = 2 K).
